# High fat and alcoholic diets increase hepatic EMH, osteolysis and spontaneous metastases by orthotopic mammary tumors

**DOI:** 10.1186/2051-1426-1-S1-P258

**Published:** 2013-11-07

**Authors:** Anand Dusad, Saraswoti Khadge, Geoffrey M Thiele, Ted R Mikuls, Michael J Duryee, Holly C Britton, Lynell W Klassen, Alicia J Dafferner, Tracy Farrell, Timothy R McGuire, Carlos D Hunter, Karen C Easterling, Barbara J O'Kane, John G Sharp, James E Talmadge

**Affiliations:** 1 Pathology & Microbiology, University of Nebraska Medical Center, Omaha, NE, USA; 2 Internal Medicine Rheumatology, University of Nebraska Medical Center, Omaha, NE, USA; 3 Genetics Cell Biology & Anatomy, University of Nebraska Medical Center, Omaha, NE, USA; 4 COP Pharmacy Practice, University of Nebraska Medical Center, Omaha, NE, USA; 5 College of Arts and Sciences, University of Nebraska Omaha, Omaha, NE, USA

## 

High-fat diets and chronic alcohol consumption (CAC) can both induce a low-grade inflammation. We report that when CAC (16.6% of total calories) is administered in combination with the Lieber-DeCarli high-fat diet an additive myeloid response is induced. This increase in inflammation included hepatic and splenic EMM as assessed by flow cytometry, immunohistochemistry and a colony forming unit-granulocyte macrophage (CFU-GM) assay. Further, an increased number of hepatic myeloid derived suppressor cells (MDSCs) were observed that were predominantly Ly6c bright. The increase in MDSCs was associated with an increased number of hepatic non-parenchymal cells. Consistent with the increased number of hepatic MDSCs and EMM was a decrease in bone marrow cellularity, MDSCs and progenitor cells (Lin-CD11b-Gr1-Sca-1+), resulting in a decrease in CFU-GM/femur. Unexpectedly, we observed demineralization and osteolytic lesions by micro computed tomography (micro CT) in all bones examined including femur, tibia, humerus and vertebral column. The low grade, chronic inflammation associated with the Lieber-DeCarli high fat diet and CAC increased the growth of orthotropic 4T1 mammary tumors, resulting in extensive bone osteolysis, demineralization, myeloplasia and lymphopenia and increased metastasis as defined by pulmonary lesions and aberrant metastatic sites including splenic, cardiac, hepatic and large and extensive lymphatic foci in addition to peritoneal effusions. The effusions were haemorrhagic with predominantly a nucleated cell infiltrate composed of bands, segs and myelocytes, supporting EMM. The osteolysis was most notable in the distal femur, proximal tibia, and vertebral spurs, associated with osteoclast channels and the demineralization appeared to be associated with areas of active myelopoiesis. We suggest that a high fat diet and CAC can increase tumor metastasis and pathology in association with myeloid progenitor mobilization and EMM resulting in increased numbers of osteoclasts, MDSCs, associated bone demineralization and suppression of T-cell frequency and function. These results support the use of combination therapy strategies that incorporate multiple molecular therapeutics that inhibit OCs, MDSCs and their associated mediators i.e. COX-2, NO.

